# The Commensal Anaerobe Veillonella dispar Reprograms Its Lactate Metabolism and Short-Chain Fatty Acid Production during the Stationary Phase

**DOI:** 10.1128/spectrum.03558-22

**Published:** 2023-03-28

**Authors:** Shi-Min Zhang, Shir-Ly Huang

**Affiliations:** a Institute of Clinical Medicine, National Yang Ming Chiao Tung University, Yangming Campus, Taipei, Taiwan; b Institute of Microbiology and Immunology, National Yang Ming Chiao Tung University, Yangming Campus, Taipei, Taiwan; University of Minnesota Twin Cities

**Keywords:** *Veillonella dispar*, short-chain fatty acids, lactate, stationary phase, anaerobe, transcriptome, propionate metabolism, acetate

## Abstract

*Veillonella* spp. are obligate, anaerobic, Gram-negative bacteria found in the human oral cavity and gut. Recent studies have indicated that gut *Veillonella* promote human homeostasis by producing beneficial metabolites, specifically short-chain fatty acids (SCFAs), by lactate fermentation. The gut lumen is a dynamic environment with fluctuating nutrient levels, so the microbes present shifting growth rates and significant variations of gene expression. The current knowledge of lactate metabolism by *Veillonella* has focused on log phase growth. However, the gut microbes are mainly in the stationary phase. In this study, we investigated the transcriptomes and major metabolites of Veillonella dispar ATCC 17748^T^ during growth from log to stationary phases with lactate as the main carbon source. Our results revealed that *V. dispar* reprogrammed its lactate metabolism during the stationary phase. Lactate catabolic activity and propionate production were significantly decreased during the early stationary phase but were partially restored during the stationary phase. The propionate/acetate production ratio was lowered from 1.5 during the log phase to 0.9 during the stationary phase. Pyruvate secretion was also greatly decreased during the stationary phase. Furthermore, we have demonstrated that the gene expression of *V. dispar* is reprogrammed during growth, as evidenced by the distinct transcriptomes present during the log, early stationary, and stationary phases. In particular, propionate metabolism (the propanediol pathway) was downregulated during the early stationary phase, which explains the decrease in propionate production during the stationary phase. The fluctuations in lactate fermentation during the stationary phase and the associated gene regulation expand our understanding of the metabolism of commensal anaerobes in changing environments.

**IMPORTANCE** Short-chain fatty acids produced by gut commensal bacteria play an important role in human physiology. Gut *Veillonella* and the metabolites acetate and propionate, produced by lactate fermentation, are associated with human health. Most gut bacteria in humans are in the stationary phase. Lactate metabolism by *Veillonella* spp. during the stationary phase is poorly understood and was therefore the focus of the study. To this end, we used a commensal anaerobic bacterium and explored its short-chain fatty acid production and gene regulation in order to provide a better understanding of lactate metabolism dynamics during nutrient limitation.

## INTRODUCTION

*Veillonella* spp. are obligate anaerobic, Gram-negative bacteria found in the human oral cavity (mainly the dorsal surface of the tongue) and in the colon (1, 2). They utilize lactate or other organic acids as their main carbon sources. Recent omics studies have indicated that *Veillonella* spp. and their metabolites, acetate and propionate (short-chain fatty acids or SCFAs), are linked to human health (3 to 6). Gut *Veillonella* have been identified as exercise-performance-enhancing bacteria in one meta-omics study; specifically, the abundance of *Veillonella* spp. and the lactate-to-propionate catabolic pathway were found to be significantly enriched in the gut microbiota of elite athletes (5). In one recent randomized study, the abundance of gut *Veillonella*, especially *V. dispar*, was found to be significantly increased in individuals taking 2 weeks of moderate-intensity continuous training, and this was associated with improved endotoxemia (7). *Veillonella* spp. have been found to be significantly depleted in the gut microbiota of patients with autism spectrum disorders (8) and children at risk of asthma (9). Furthermore, these alterations are associated with a reduced level of acetate in feces (9). However, *Veillonella* spp. have also been found to be associated with diseases such as autoimmune hepatitis (10). In particular, *V. dispar* was identified as a robustly disease-associated bacterium (10), although causality was not established. The differences between these studies investigating the role of *V. dispar* in human physiology strengthen the need for a deeper understanding of *Veillonella*.

*Veillonella* spp. ferment lactate into acetate and propionate, which are SCFAs (11, 12). SCFAs play essential roles in human physiology, such as maintaining colonic immune homeostasis (13, 14), appetite regulation (15), and regulation of the host’s energy balance (16). The gut environment is a dynamic environment with a fluctuating nutrient level that is determined by the host’s dietary pattern, such as fasting. Nutrient limitation present in the gut lumen restricts the growth of gut microbes and directs their growth patterns into a stationary phase (17), and this has been shown to induce a microbial metabolite profile with increased SCFAs (18 to 21). Hence, it is crucial to study *Veillonella* lactate metabolism during the stationary phase in order to obtain an understanding of the physiology of these anaerobic bacteria during nutrient limitation. It is known that in the stationary phase, aerobic bacteria will slow down their growth, reduce their metabolic activity, and exploit alternative nutrient sources or even use bacterial internal storage (22, 23). The adaption strategies used by starved bacteria include scavenging environmental nutrients by upregulation of high-affinity transporters (24), and the rerouting of metabolic pathways in order to flexibly utilize metabolic intermediates (25, 26). Further studies are needed in order to understand the adaptation strategies of anaerobic bacteria.

Here, we investigated the global transcriptome and metabolic profile of a representative commensal anaerobic bacterium, Veillonella dispar ATCC 17748^T^, during the growth from the log phase to stationary phase. *V. dispar* is commonly isolated from human specimens and has been detected during several metagenomic analyses (1, 27, 28). This study integrates the analysis of major metabolites involved in lactate catabolism and the bacterial transcriptome in order to provide a better understanding of how commensal anaerobes respond to the nutrient limitation during the stationary phase.

## RESULTS

### The growth, metabolite production, and viability of *V. dispar* during anaerobic cultivation in TYL medium and TY medium.

**FIG 1:**
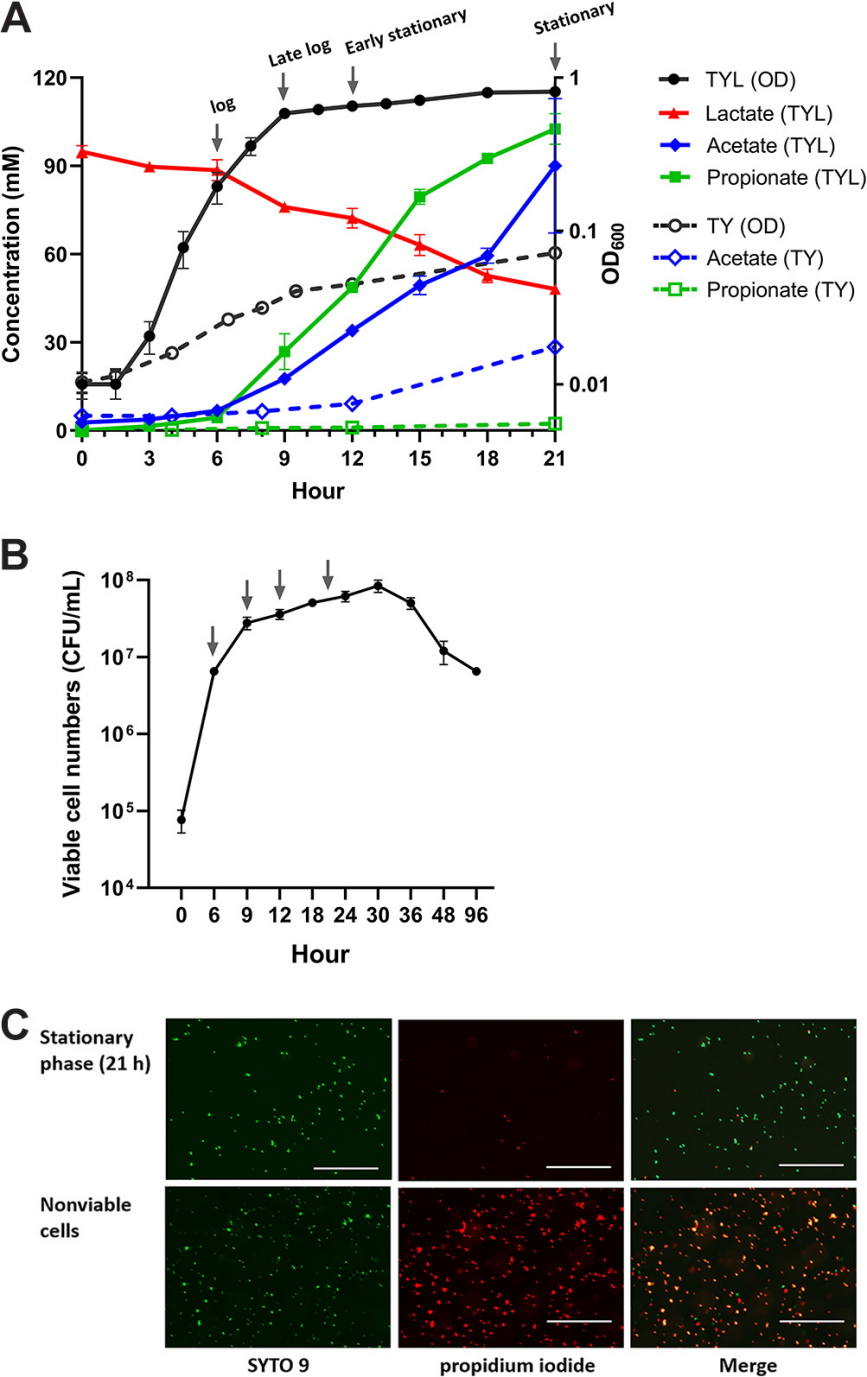
Growth, metabolite production, and viability of *V. dispar* during anaerobic cultivation in TYL medium. (A) Growth and extracellular metabolites production of *V. dispar* cultivated in the TYL and TY (control) medium, 37°C, anaerobically. The arrows from left to right indicate the four growth phases and harvesting times: log phase (6 h), late log phase (9 h), early stationary phase, and stationary phase (21h). (B) Viable cell counts (CFU/mL) of *V. dispar* during the anaerobic cultivation in TYL medium. The arrows indicate the four phases corresponding to panel A. (C) Viability of cells grown at 21 h cultivated in TYL medium anaerobically. All cells were stained green with SYTO 9; only nonviable cells were stained red with propidium iodide with the scale bar of 50 μm. A representative image of three biological replicates is shown. OD_600_, optical density of bacteria suspension at wavelength 600 nm. Data are mean ± SEM of three biological replicates; unpaired two-tailed *t* test was used for statistical analysis.

*V. dispar* was inoculated into the tryptone-yeast-lactate (TYL) medium with an initial optical density (O.D.) of 0.01 and was found to have a short lag phase. Sequentially, *V. dispar* underwent a log phase, then late log phase, early stationary phase, and stationary phase (Fig. 1A). For the following experiments, *V. dispar* was harvested at 6 h, 9 h, 12 h, and 21 h, which were operationally defined as log phase, late log phase, early stationary phase, and stationary phase, respectively (Fig. 1A). Bacteria harvested between 3 and 6 h with the same doubling time of 56 min were all growing in the log phase. At the stationary phase (21h), the cells remained mostly viable (Fig. 1B and C), and they remained mostly viable until 36 h (Fig. 1B).

*V. dispar* encodes only l-lactate dehydrogenase and therefore utilizes only l-lactate (29). The TYL medium contains 107 mM dl-lactate, a racemic mixture, with an initial detected concentration of 95 mM by high-pressure liquid chromatography (HPLC) analysis. During anaerobic growth, the lactate in the TYL medium was continually consumed, and the concentration was found to have been reduced to 48.5 mM, probably mostly d-lactate, at 21 h. Thus, *V. dispar* encounters l-lactate limitation at some point during the stationary phase. Acetate production was stable during early cultivation but significantly increased from 18 h to 21 h (stationary phase). Propionate production slowed down from 15 h onwards (Fig. 1A). As a result, the propionate/acetate ratio in the medium changed over time. It was 1.6 during the early cultivation (6 to 15 h) and 0.6 during the late cultivation (15 to 21 h).

We cultivated *V. dispar* in the tryptone-yeast (TY) medium with an initial O.D. of 0.01 as a control. *V. dispar* grew poorly in the TY medium, with the O.D. only reaching 0.072 at 21 h. *V. dispar* cultivated in the TY medium produced about 1/50 of the propionate and 1/3 of the acetate compared to when it was grown in the TYL medium during late growth phase (Fig. 1A).

### *V. dispar* exhibits an altered lactate metabolic profile during the stationary phase.

In order to investigate the metabolic profile of *V. dispar*, we prepared cells of *V. dispar* that had been harvested during the four different growth phases, and these were analyzed to establish the major metabolites produced by lactate metabolism. Our results revealed that *V. dispar* catabolized l-lactate and secreted various metabolites, specifically pyruvate, acetate, and propionate, into the environment. The proposed metabolic pathway and intermediates are shown in Fig. S1 in the supplemental material.

**FIG 2:**
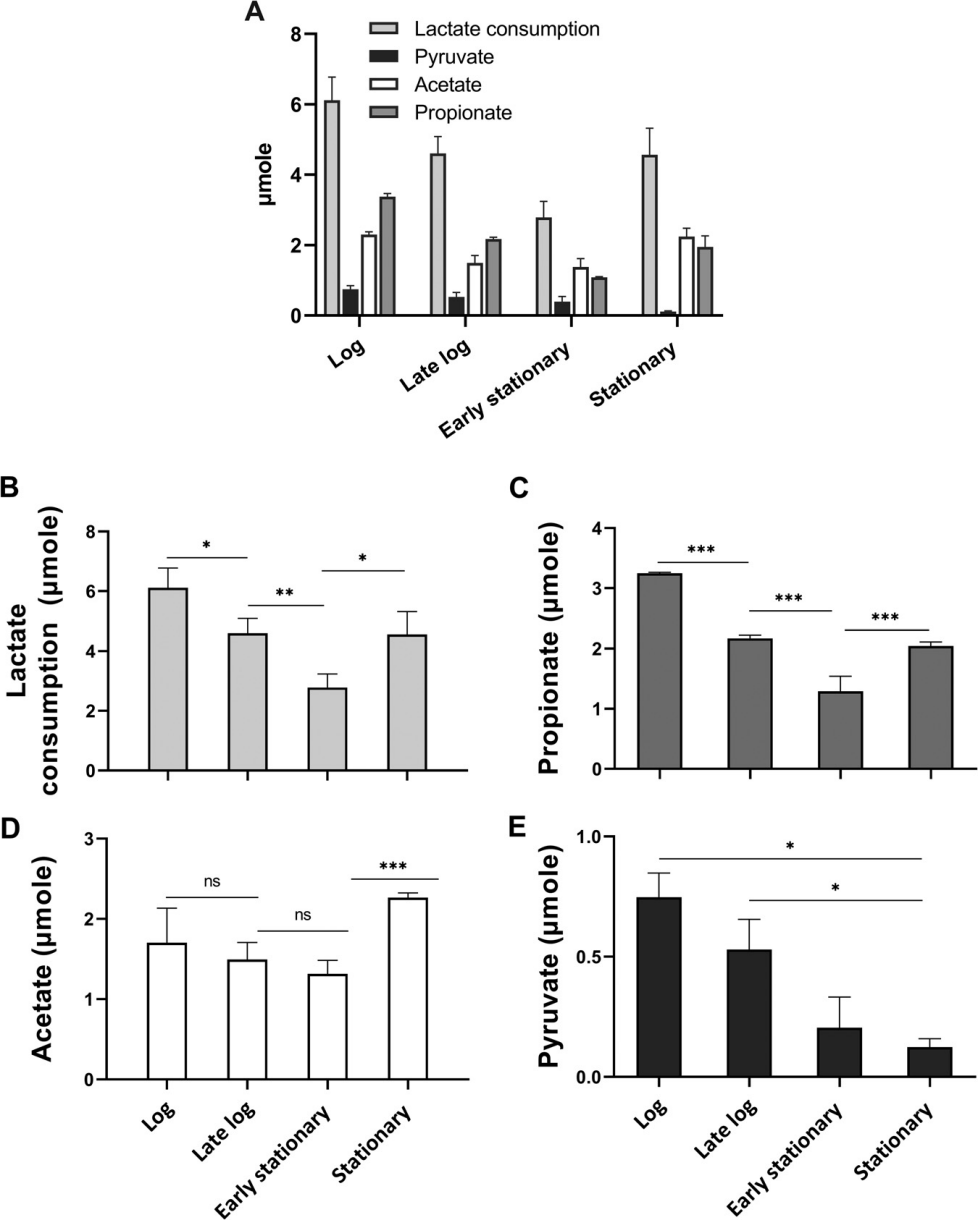
Lactate consumption and major metabolites secretion from *V. dispar* cells harvested from the four growth phases. The lactate metabolic profile of *V. dispar* at each growth phase is summarized in panel A and is separately shown as (B) lactate consumption and secreted amounts of (C) propionate, (D) acetate, and (E) pyruvate. Data are mean ± SEM of three biological replicates; unpaired two-tailed *t* test was used for statistical analysis. *, *P* < 0.05; **, *P* < 0.01; ***, *P* < 0.001; ns, not significant.

We have illustrated the lactate metabolic profiles of *V. dispar* cells over the four phases (Fig. 2A), and from this we identified that major metabolite production via lactate fermentation is redistributed in the stationary phase. First, lactate catabolic activity and propionate production were both significantly decreased during the early stationary phase but then were partially restored during the stationary phase (Fig. 2B and C). Acetate production was relatively stable but did show a significant increase during the stationary phase (Fig. 2D). This increase in acetate production is consistent with the results of the TYL medium metabolite analysis, which showed a sudden increase in acetate level during the stationary phase (Fig. 1A). Second, the propionate/acetate production ratio, the ratio of the two main metabolites, was lowered during the stationary phase. It was 1.5, 1.5, 0.8, and 0.9 during the log phase, late log phase, early stationary phase, and stationary phase, respectively. Third, pyruvate secretion was also significantly decreased during the stationary phase but with a gradual transition (Fig. 2E).

**FIG 3:**
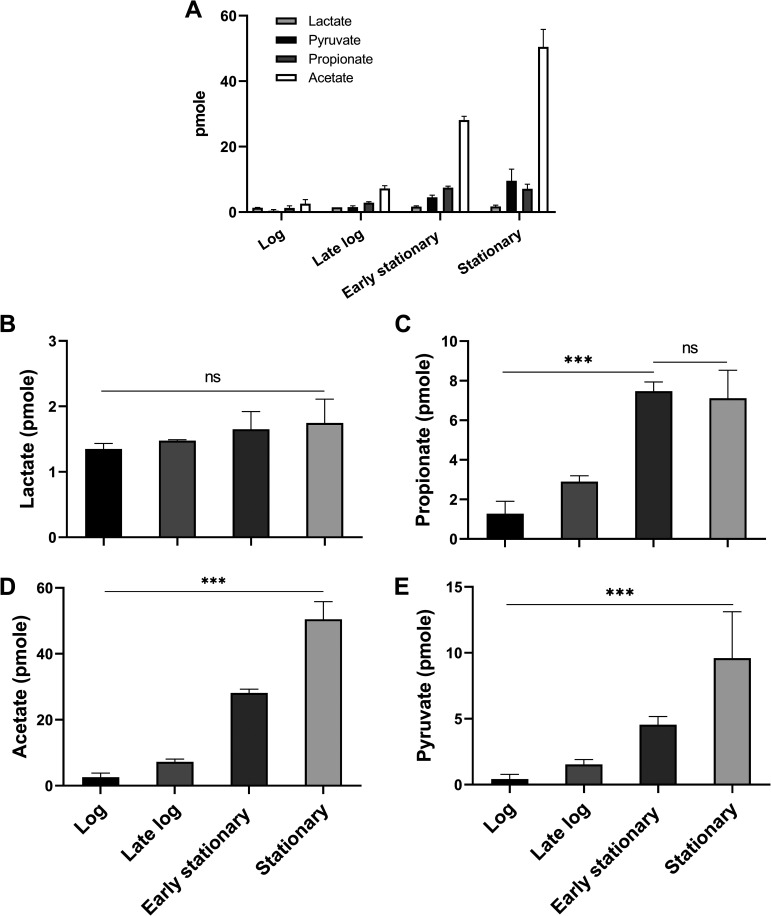
Intracellular metabolite analysis of reacted cells over the four growth phases. Intracellular metabolite content of cells harvested at each growth phase after lactate catabolic reaction is summarized in panel A. The measured metabolite includes (B) lactate, (C) propionate, (D) acetate, and (E) pyruvate. The amount of metabolite was extracted from 10^8^ cells. Extracellular lactate consumption and SCFA secretion are shown in Fig. 2. Data are mean ± SEM; unpaired two-tailed *t* test was used for statistical analysis. ***, *P* <0.001.

We further analyzed the intracellular metabolite profile of *V. dispar* cells after they underwent lactate catabolism; we measured various metabolites, including lactate, propionate, acetate, and pyruvate (Fig. 3A). Here, these cells are referred to as reacted cells. The targeted intracellular metabolite levels extracted from 10^8^ reacted cells were low compared to the secreted extracellular metabolite levels (from the same reaction mixture). Reacted cells during the log phase had low targeted intracellular metabolite levels compared to the other phases (Fig. 3A). Reacted cells over the four growth phases had similar and low intracellular lactate levels (Fig. 3B). When intracellular propionate was examined, it was found to increase from the log phase to early stationary phase (Fig. 3C). Reacted cells during the log phase had low intracellular acetate and pyruvate levels, and both of these were significantly increased during the stationary phase (Fig. 3D and E).

### *V. dispar* shows dynamic gene expression when growing from log phase to stationary phase.

**FIG 4:**
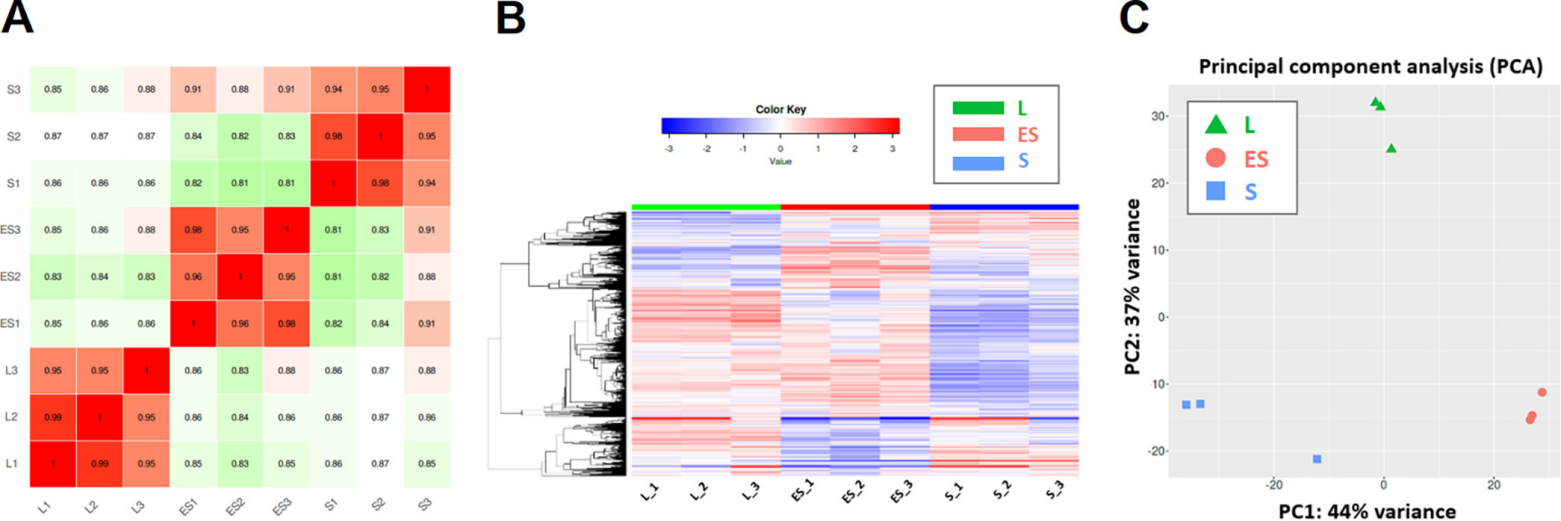
Gene expression profile of *V. dispar* during the anaerobic growth. The transcriptomes of *V. dispar* harvested during log phase (L), early stationary phase (ES), and stationary phase (S). (A) Correlation matrix of each comparison pair labeled with Pearson’s correlation coefficient. (B) Heatmap of hierarchical cluster of all genes from all samples. (C) Principal-component analysis (PCA) of the transcriptomes from the three growth phases.

To investigate the gene expression of *V. dispar* during the growth from log phase to stationary phase, we used the RNA-seq method to evaluate the transcriptome of *V. dispar* during the log phase, early stationary phase, and stationary phase (*n* = 3 in each group; total *n* = 9). The correlation coefficients for comparison pairs within the same biological group were generally >0.95, indicating that there was good biological reproducibility of the samples within the same group (Fig. 4A).

On hierarchical clustering analysis, the transcriptomic profiles of *V. dispar* harvested during the log phase, early stationary phase, and stationary phase were distinct (Fig. 4B), which is consistent with the result of principal-component analysis (Fig. 4C).

An analysis of the differentially expressed genes (DEGs) at the transcript level used log phase as a control. Our results show that 149 genes were upregulated (at least >1.5 log_2_ fold change), and 201 genes were downregulated during the early stationary phase. On the other hand, 90 genes were upregulated and 375 genes were downregulated during the stationary phase (Fig. S2). The genes upregulated between the early stationary phase and the stationary phase showed little overlap (Fig. S2). These DEGs were then subjected to KEGG enrichment analysis, which revealed that propionate metabolism, gluconeogenesis, several amino acid metabolisms/biosyntheses, ribosome genes, and nitrogen metabolism were differentially expressed (Table 1).

**TABLE 1:** Enrichment analysis of the differentially expressed genes

KEGG term	Enrichment ratio	*P* value
Early stationary phase vs log phase		
Ribosome	0.51	<0.01
Propanoate metabolism	0.42	<0.01
Carbon metabolism	0.25	<0.01
Metabolic pathways	0.11	<0.01
Biosynthesis of secondary metabolites	0.15	<0.01
Biosynthesis of amino acids	0.17	0.013
Microbial metabolism in diverse environments	0.17	0.015
Two-component system	0.31	0.015
Alanine, aspartate, and glutamate metabolism	0.35	0.018
Biosynthesis of antibiotics	0.15	0.022
Nitrogen metabolism	0.35	0.031
Histidine metabolism	0.40	0.043
One carbon pool by folate	0.40	0.043
Glycolysis/gluconeogenesis	0.29	0.047
Methane metabolism	0.36	0.049
Stationary phase vs early stationary phase		
Metabolic pathways	0.17	<0.01
Alanine, aspartate, and glutamate metabolism	0.70	<0.01
Biosynthesis of antibiotics	0.21	0.013
Purine metabolism	0.33	0.015
Biosynthesis of secondary metabolites	0.17	0.036
Propanoate metabolism	0.38	0.036
Stationary phase vs log phase		
Ribosome	0.83	<0.01
Biosynthesis of secondary metabolites	0.21	0.019
Biosynthesis of amino acids	0.26	0.019
Biosynthesis of antibiotics	0.24	0.019
Phenylalanine, tyrosine, and tryptophan biosynthesis	0.47	0.027
Purine metabolism	0.33	0.047
Valine, leucine, and isoleucine biosynthesis	0.50	0.086

### Propionate metabolism (the propanediol pathway) was downregulated during the early stationary phase.

**FIG 5:**
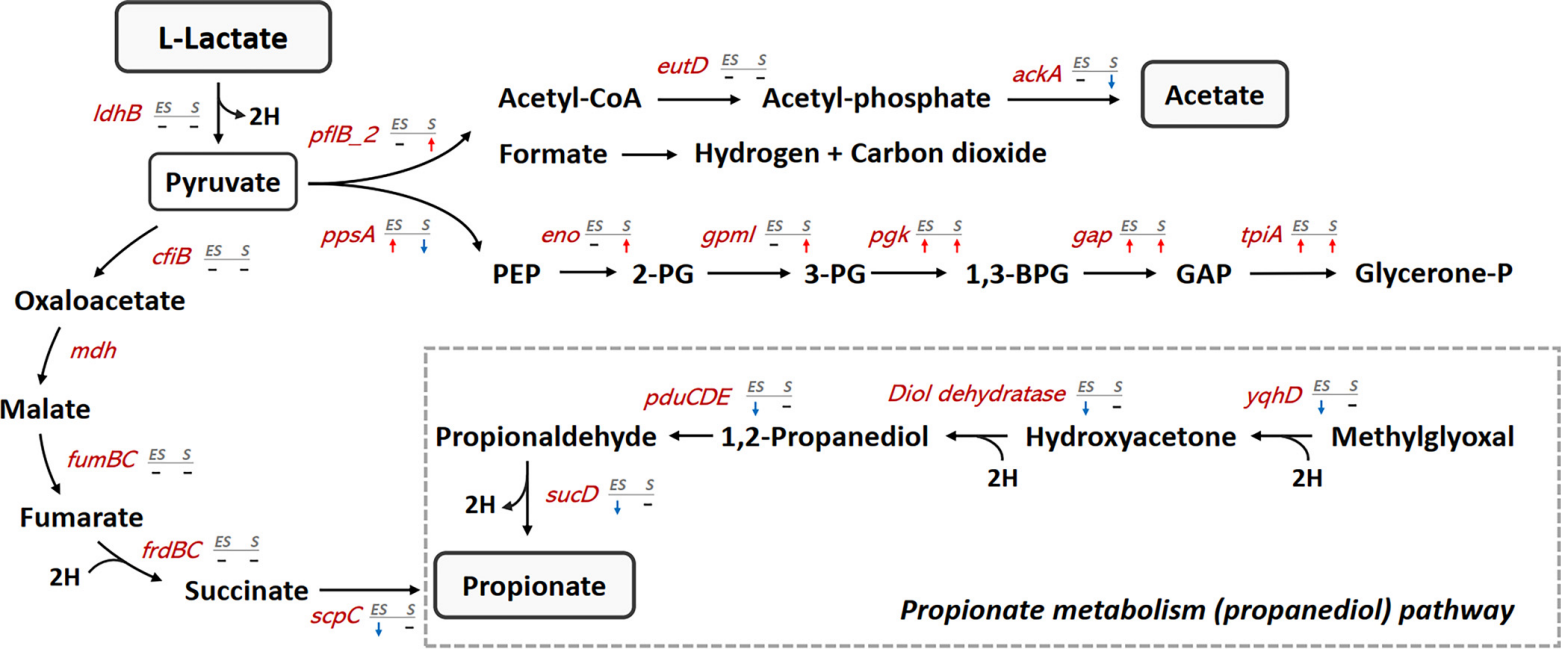
Regulation of the genes associated with lactate/propionate metabolism. The pathway and transcript level of lactate/propionate metabolism-associated genes are illustrated in the schematic diagram. Metabolite name is shown in black and gene name in red. The red arrow indicates significant upregulation (≥1.5 log_2_ fold change) of gene expression compared to that in the log phase; the blue arrow indicates significant downregulation (≤1.5 log_2_ fold change); dash indicates insignificant transcript level change. PEP, phosphoenolpyruvate; 2-PG, 2-phosphoglycerate; 3-PG, 3-phosphoglycerate; 1,3-BPG, 1,3-bisphosphoglycerate; GAP, glyceraldehyde 3-phosphate; Glycerone-P, glycerone phosphate; *ldhB*, l-lactate dehydrogenase; *cfiB*, pyruvate carboxylase; *fumB*, fumarate hydratase, class I; *fumC*, fumarate hydratase, class II; *frdB*, fumarate reductase iron-sulfur subunit; *frdC*, fumarate reductase, cytochrome b subunit; *scpC*, succinyl-CoA: acetate CoA-transferase; *pflB_2*, formate acetyltransferase; *eutD*, phosphate acetyltransferase; *ackA*, acetate kinase; *ppsA*, phosphoenolpyruvate synthase; *eno*, enolase; *gpmI*, 2,3-bisphosphoglycerate-independent phosphoglycerate mutase; *pgk*, phosphoglycerate kinase; *gap*, glyceraldehyde 3-phosphate dehydrogenase; *tpiA*, triosephosphate isomerase; *yqhD*, NADP-dependent alcohol dehydrogenase; *pduC*, propanediol dehydratase large subunit; *pduD*, propanediol dehydratase medium subunit; *pduE*, propanediol dehydratase small subunit; *sucD*, propionaldehyde dehydrogenase.

The selected DEGs and their fold change in transcript levels are shown in Table S1. Our finding shows that most *V. dispar* genes within the lactate fermentation pathway were not differentially expressed during anaerobic growth (Fig. 5). However, our results did identify that propionate metabolism (the propanediol pathway) was downregulated during the early stationary phase (Table 1). The genes involved in propionate metabolism that were differentially expressed included *yqhD* (alcohol dehydrogenase), *pduCDE* (propanediol dehydratase), and *sucD* (propionaldehyde dehydrogenase), all of which were significantly downregulated during the early stationary phase (Fig. 5) and then were upregulated again during the stationary phase compared to the early stationary phase.

### Pyruvate-utilization-related genes were upregulated in the early stationary and stationary phases.

Genes associated with pyruvate utilization, including *pflB*, *poxB*, and *ppsA*, were regulated during anaerobic growth. The gene *pflB* (pyruvate formate-lyase) acts as an upstream regulator of acetate production and was significantly upregulated during the stationary phase. Its upregulation explains the increase in acetate production by *V. dispar* during the stationary phase (Fig. 2D) and the decrease in ratio of propionate/acetate production during the stationary phase. The genes *poxB* (pyruvate dehydrogenase [quinone]) and *ppsA* (phosphoenolpyruvate synthase) were both significantly upregulated during the early stationary phase but downregulated during the stationary phase (Fig. 5). In addition, our DEG enrichment analysis identified that gluconeogenesis was upregulated during the early stationary phase (Table 1). The genes involved in gluconeogenesis that were differentially expressed include *ppsA*, *eno*, *gpmI*, *pgk*, *gap*, and *tpiA*. Of these genes, *eno* and *gpmI* were significantly upregulated during the early stationary phase; *pgk*, *gap*, and *tpiA* were upregulated during both the early stationary and stationary phases (Fig. 5).

### Other differentially expressed genes of metabolic importance.

In addition to propionate metabolism and gluconeogenesis, the gene involved in metabolism/biosynthesis of several amino acids, in nitrogen metabolism, in biotin biosynthesis, and a number of ribosomal proteins were also modulated during the various growth phases. Histidine biosynthesis genes were upregulated during the early stationary phase (Fig. S3) with the upregulated genes, including *hisA*, *hisB*, *hisC*, *hisD*, *hisE*, *hisF*, *hisG*, and *hisI*; phenylalanine, tyrosine, and tryptophan biosynthesis genes were downregulated during the stationary phase (Fig. S3) with the downregulated genes, including *trpC*, *trpE*, *aroA*, *aroB*, *aroC*, *aroE*, *aroF*, *aroK*, and *pheA*; valine, leucine, and isoleucine biosynthesis genes were downregulated during the stationary phase (Fig. S3) with the downregulated genes, including *leuA*, *leuB*, *dmdA*, *tdcB*, and *ilvD*; nitrogen metabolism genes were upregulated during the early stationary phase (Table S1) with the upregulated genes, including *norB* (nitric oxide reductase), *glnA* (glutamine synthetase), *gltB* (ferredoxin-dependent glutamate synthase), and *narK* (nitrite facilitator); biotin biosynthesis genes were significantly upregulated during the stationary phase (Table S1) with the upregulated genes, including *bioB*, *bioD*, *bioF*, *bioK*, and *bioW*; and finally, various ribosomal protein were downregulated during the early stationary and stationary phase (Table S1).

## DISCUSSION

This study investigated the lactate metabolism by *V. dispar* during various different growth phases, with a focus on the bacteria's gene expression and its major metabolites. In particular, the transition states between log phase and stationary phase, and between late log phase and early stationary phase, were studied. In the present study, *V. dispar* was cultivated in the TYL medium, which contains sodium dl-lactate as a carbon source. The growth of *V. dispar* in TYL medium using sodium l-lactate alone and sodium dl-lactate are indistinguishable. *V. dispar* was also cultivated in the TY medium as a control. *V. dispar* grew poorly in the TY medium and produced only a little propionate and about one-third of the acetate at 21 h compared to growth in TYL medium (Fig. 1A). This supports the hypothesis that the supplemented lactate in the TYL medium was the main carbon source. We harvested *V. dispar* during the various growth phases and assessed lactate consumption and the release of SCFAs such as acetate and propionate in the medium. The use of washed cells allowed us to evaluate metabolic activity of *V. dispar* during each specific growth phase. The bacterial cultivation and metabolic activity experiments were conducted in an environment containing 5% hydrogen. Previous studies have indicated that the presence of hydrogen has an insignificant effect on *Veillonella* lactate fermentation (11, 30).

Microbes respond to nutrient limitation by reprogramming their metabolisms, such as by increasing fermentation or by decreasing aerobic respiration (22, 23). Our study reveals that *V. dispar* undergoes alteration in its lactate metabolic profiles and its transcriptome as the bacteria moves from log phase to stationary phase. Over this time, the growth of *V. dispar* slowed beginning with the early stationary phase, and the nutrient levels in the medium moved toward a famine state. We observed several notable features within the altered lactate metabolic profile at the stationary phase. These included the dynamic lactate catabolic activity, active propionate production, a decreased propionate:acetate ratio, and a significant decrease in pyruvate secretion. First, lactate catabolic activity and propionate production were significantly decreased during the early stationary phase but were then partially restored during the stationary phase (Fig. 2B and C). A possible explanation for this phenomenon is that *V. dispar* may have an increased substrate affinity during the stationary phase. Second, the main metabolites associated with the propionate:acetate ratio decreased gradually during anaerobic growth, which is consistent with the results of the metabolite analysis of TYL medium (Fig. 1A). Though propionate production was partially restored during the stationary phase, the acetate production was significantly increased at the same time. We also showed that the intracellular acetate level of the reacted cells during the stationary phase was higher than that during the log phase (Fig. 3D). The increased levels of both extracellular acetate and intracellular acetate may be explained by the upregulation of pyruvate-formate lyase during the stationary phase; this change will have directed the carbon flow into acetate production (Fig. 5). Third, the significant decrease in pyruvate secretion during the stationary phase (Fig. 2E) may be the result of utilization of the key intermediate pyruvate by *V. dispar* for both catabolism and anabolism during the early stationary phase and stationary phase. Our intracellular metabolites analysis found that the intracellular pyruvate level of the reacted cells during the stationary phase was greatly increased compared to the log phase (Fig. 3E). Our transcriptomic data showed that gluconeogenesis was upregulated during the early stationary phase (Table 1). Although *Veillonella* spp. lack glycolytic activity due to a lack of hexokinase (31), *V. parvula* M4 is able to convert lactate into phosphoenolpyruvate (32), which suggests that the gluconeogenesis pathway in *V. dispar* has the possibility of still being able to function because a number of genes other than hexokinase are conserved. As pyruvate accumulates inside the cell during the stationary phase (Fig. 3E), the upregulation of gluconeogenesis should theoretically provide an alternative metabolic route for cells under nutrient limitation stress. Overall, our findings reveal that *V. dispar* redistributes its production of major metabolites during the stationary phase, from producing more propionate than acetate (1.5:1) during the log phase to producing a nearly equal amount of propionate and acetate (1:0.9) during the stationary phase. Acetate and propionate (both SCFAs) play important roles in human physiology, and therefore our results provide helpful information when the roles of *Veillonella* spp. in human physiology are being explored in the future. Specifically, the growth phase of the organisms within the human gut needs to be given seriously consideration.

We found that *V. dispar* has an altered lactate metabolic profile during the stationary phase, and to further explore this the gene regulation of related metabolic pathways needs to be elucidated. Transcriptomics was used to investigate *V. dispar* gene expression during the log phase, early stationary phase, and stationary phase. Our results provide information on the transcript levels of almost all of the genes encoded by V. dispar and reveals that *V. dispar* exhibited a dynamic transcriptome during growth from log phase to stationary phase. Both hierarchical clustering analysis (Fig. 4B) and principal-component analysis (Fig. 4C) suggest that *V. dispar* is reprogramming its global gene expression to adjust to the changing environmental conditions; this largely involves differentially expressed genes related to lactate metabolism, propionate metabolism (the propanediol pathway), citrate lyase, nitrogen metabolism, biotin biosynthesis, and amino acid biosynthesis. These findings agree with a previous study showing that E. coli is able to reshape its proteome during the stationary phase; these changes mostly involved differentially expressed proteins relating to metabolism (17). When lactate-metabolism-associated pathways are explored, the genes associated with propionate metabolism (the propanediol pathway) were found to be significantly downregulated during the early stationary phase, which may explain the significant decrease in propionate production during the early stationary phase. *Veillonella* spp. are known to use the methylmalonyl pathway for lactate fermentation; however, recent metagenomic analysis has indicated that *Veillonella* spp. may also use the propanediol pathway for propionate production (33). Our findings suggest that the diversity of propionate metabolism routes within *V. dispar* needs to be further investigated in order to obtain a better understanding of the dynamics of propionate production profile during the log phase, early stationary phase, and stationary phase.

In addition to lactate-metabolism-associated genes, genes associated with amino acid biosynthesis, nitrogen metabolism, biotin biosynthesis, and the ribosome were found to be also regulated during growth. Biotin is an essential cofactor for biotin-dependent enzymes, including pyruvate carboxylase and methylmalonyl-CoA decarboxylase (34), which are involved in catalyzing the formation of propionyl-CoA from methylmalonyl-CoA. The upregulation of such genes during the stationary phase seems to support the hypothesis that *V. dispar* is trying to restore lactate catabolic activity during the stationary phase. The downregulation of ribosome-associated genes during the stationary phase is consistent with previous studies showing that bacteria-downregulate genes were associated with rRNA and ribosomal protein biosynthesis during the stationary phase under amino-acid starvation (23), or when there is reduced protein synthesis (35).

Understanding the dynamic metabolic profiles of gut microbes is important because the human gut lumen is an ever-changing environment. The gut microbial community in both mice and humans undergoes daily oscillations that are affected by the host’s feeding behavior (36). For example, intermittent fasting is an emerging, clinically meaningful regime of diet intervention that directly affects the gut microbial community via the regulation of nutrient sources, which can then alter host–microbe interactions. It has been found to have strong disease-modifying effects on various chronic diseases, including obesity, diabetes, and cardiovascular disease (37 to 39). Individuals who undertake an intermittent fasting program experience long-term starvation, which significantly reduces the nutrient sources available to the gut microbial community. The composition, function, and metabolite profile of the gut microbial community are altered after the host starts an intermittent fasting program (18 to 21, 40, 41). The importance of understanding the dynamic metabolic profiles of gut microbes using a representative emerging commensal bacterium as a model has been demonstrated by this study. The roles of *Veillonella* spp. in modulating the host’s physiology were not able to be explored in detail until recent omics studies could be linked to human health (5). However, our understanding of *Veillonella* physiology still remains relatively limited. In this study, we illustrated that metabolic profiling and transcriptomics can be used to explore *V. dispar* growth. Our findings have provided a fundamental understanding of the *Veillonella* metabolic profile and of its gene expression, particularly during the stationary phase, and may now be applied to human gut microbiome studies. For example, individuals who have undertaken an intermittent fasting program have been found to have higher fecal SCFA levels (18 to 21, 40, 41). Our findings show that *V. dispar* has increased acetate production during the stationary phase, and this may partially explain this phenomenon. We have only discussed lactate catabolic activities and the representative SCFA production, and obviously there are other microbial metabolites that need to be further investigated. Understanding the microbial responses under nutrient limitation provides important knowledge regarding the bacterial physiology of commensal gut anaerobic bacteria.

## MATERIALS AND METHODS

### Bacterial strain and culture condition.

*V. dispar* ATCC 17748^T^ was cultured in TYL medium (6, 42). TYL medium contains the following (g L^−1^): tryptone (BD Difco), 5.0; yeast extract (BD Difco), 3.0; sodium dl-lactate (Sigma), 12.0; K_2_HPO_4_ (Merck), 4.672; KH_2_PO_4_ (Sigma), 3.154. The pH value was adjusted to 7.0 at room temperature by NaOH. TY medium contains the same ingredients as TYL medium except for the absence of sodium dl-lactate and was used as a control medium for studying the *V. dispar* growth. All culture medium and buffers used in the following experiments were de-aerated by at least 72 h equilibration in an anaerobic chamber. Bacteria were cultivated in an anaerobic glove chamber (ThermoFisher Scientific; N_2_, 85%; CO_2_, 10%; H_2_, 5%). Bacterial growth was measured by monitoring the OD of the culture medium at 600 nm by using a spectrophotometer. The contamination by other organisms was excluded by observing colony morphology macroscopically and Gram staining microscopically.

The lactate and SCFAs in the medium were extracted using the protocol of a previous study (43). In brief, each sample had 0.2 M succinic acid added as an internal standard, which was followed by the addition of 100 μL of concentrated hydrochloric acid. The samples were then mixed with 5 mL of diethyl ether (TCI) for 20 min. Next, the upper phase was transferred to another extraction tube and mixed with 500 μL of 1 M NaOH solution for 20 min. The aqueous phase was collected, and finally, 100 μL of concentrated HCl was added.

### Metabolic activity.

**(i) Resting cell preparation.** An overnight culture of *V. dispar* was subcultured in a flask containing TYL medium, and the initial OD was adjusted to 0.01 ± 0.005. The bacterial culture (150 mL) was incubated statically at 37°C in an anaerobic incubator. *V. dispar* grown in TYL medium was sampled at different cultivation times by centrifugation (4,000 *g*, 20 min, 4°C), then the cells were washed twice. The washing buffer contained 75 mM potassium chloride, 75 mM sodium chloride (Sigma), and 2 mM magnesium chloride (Merck) in 2 mM potassium phosphate buffer (4°C, pH 7.0). Bacterial cultivation, washing, and resuspending were all conducted in an anaerobic glove box.

**(ii) Lactate catabolism metabolic activity.** The analysis of lactate catabolism metabolic activity was carried out as described previously (6), with some modifications. Cell pellets of *V. dispar* harvested at different cultivation times were resuspended in 40 mM potassium phosphate buffer (pH 7.0), and the optical density of bacterial suspension was adjusted to OD 1.0 ± 0.05. The catabolic reaction was initiated by mixing 990 μL bacteria suspension and 10 μL of 1.6 M stock solution of sodium lactate to make the final concentration of sodium lactate of 16 mM. The reaction mixtures (1 mL) were incubated at 37°C in an anaerobic incubator for 60 min and terminated by adding 10 μL of 6 N perchloric acid. In brief, 16 μmol of sodium lactate (8 μmol of sodium l-lactate) was added into the reaction mixture, and the lactate consumption (μmole) and metabolites secretion (μmole) were analyzed. The reaction mixtures were centrifuged (10,000 *g*, 3 min, 4°C), and the supernatant was collected and filtered through a nylon membrane (0.22 μm; Dikma). The supernatant was then analyzed using a HPLC-PDA (photodiode array) system (Shimadzu LC-2030, Tokyo) to measure lactate consumption and the presence of various metabolic products (pyruvate, acetate, and propionate). The pellet was further analyzed in order to measure its intracellular metabolite content.

**(iii) Intracellular metabolite analysis.** The pellet after lactate catabolic reaction was collected and then washed twice with 0.85% saline solution. The intracellular metabolites were extracted using the cold methanol method (44). In brief, the pellet was resuspended in 1 mL of absolute methanol (–80°C) and then centrifuged (20,000 *g*, 5 min, 4°C). The supernatant was transferred to another clean tube. The extracted pellets were extracted again with 0.5 mL of methanol (–80°C). The intracellular metabolites of the pellets were dissolved in 1.5 mL of methanol. The samples were sent to the mass spectrometer center, National Yang Ming Chiao Tung University Instrumentation Resource Center for analysis using a LC-QTof system (Waters ACQUITY UPLC).

### Bacteria viability assay.

**(i) Plate counting.** Samples of the bacterial cultures were collected, centrifuged (4,000 *g*, 7 min, 4°C), and then the supernatant was discarded. The pellet was resuspended in TYL medium and serially diluted to an appropriate concentration. Thereafter, 100 μL of the diluted bacterial suspension was evenly spread on an agar plate, which was followed by incubation in an anaerobic chamber for 48 h at 37°C. Plates with colony numbers between 30 and 300 were chosen for colony-forming unit (CFU) calculation. The final concentration of CFU/mL was calculated as the number of counted colonies multiplied by the number of times the bacterial suspension was diluted, and then multiplied by ten, as only 100 μl of bacterial suspension was added to the agar.

**(ii) Fluorescent staining.** A LIVE/DEAD BacLight Bacterial Viability kit (Thermo Fisher Scientific) was used to determine cell viability. The cell viability assay followed the recommended protocol from the manufacturer. The 2× fluorescent dye working solution was prepared by mixing 2.5 mL component A (SYTO 9) and 2.5 mL component B (propidium iodine) according to the manufacturer’s instructions. This is optimized for live/dead cell contrast.

Bacteria in the stationary phase (grown at 21 h) were harvested, centrifuged (10,000 *g*, 3 min, 4°C) and washed twice with water. For preparation of dead cells, bacteria in the log phase were harvested and washed with 75% ethanol. Cell pellets were resuspended in water by pipetting, and the cell concentration was adjusted to an optical density of 0.25. Next, 100 μL cell suspension and 100 μL fluorescent dye were mixed and incubated in the dark at 25°C for 15 min. For fluorescence microscopy, 5 μL final solution was transferred to a glass slide, covered with an 18-mm square coverslip, sealed with nail polish, and then imaged using an automated fluorescence microscope (Olympus BX63). SYTO 9 fluorescence was detected with the FITC filter cube; propidium iodine fluorescence was detected with the TRITC filter cube.

### HPLC-PDA analysis.

A HPLC-PDA system (Shimadzu LC-2030, Tokyo) was used in this study. The autosampler was set at 10°C. YMC-Triart C18 (250 mm × 4.6 mm i.d.; 5 μm) was used and protected by a guard column. To measure lactate, pyruvate, acetate, and propionate metabolic activity, the column oven temperature was set at 25°C, and the mobile phase consisted of 93.5% solution A (20 mM NaH_2_PO_4_ in reagent-grade water, pH 2.2 adjusted by phosphoric acid) and 6.5% solution B (LC-grade acetonitrile, Merck) (43). The flow rate started at 0.1 mL/min and was gradually increased to 1.0 mL/min until analysis time reached 15 min. The overall analysis time was 25 min.

### Transcriptome analysis.

Bacteria were harvested by centrifugation (15,000 *g* for 1 min, 25°C). Total RNA was extracted using a Presto mini RNA bacteria kit according to the manufacturer’s instructions. The concentration of each extracted RNA sample was determined by using a Qubit fluorometer. The purity and integrity were assessed using a NanoPhotometer and an Agilent FA5200, respectively. For the RNA sequencing, cDNA libraries were prepared using the Illumina Ribo-Zero Plus rRNA Depletion kit according to the manufacturer’s instructions. Illumina pair-end reads (150-bp) RNA-seq were performed on an MiSeq platform by HGT Co. (Taiwan). The reads were trimmed and filtered for data quality control using a Phred score of 30. RNA-seq reads were mapped against the *V. dispar* ATCC 17748^T^ reference genome. The raw counts were normalized using the trimmed mean of M values (TMM) method. The normalized gene matrix was analyzed using iDEP (45), a website providing integrated RNA-seq data analysis applications. Differential expressed genes analysis, principal-component analysis (PCA), and hierarchical clustering were performed using this web application. Hierarchical clustering was carried out with the average linkage method by correlation distance. KEGG enrichment pathway analysis was performed with KOBAS 2.0 (46).

### Data availability.

The raw sequence data have been submitted to the Sequence Read Archive (SRA) under the BioProject number PRJNA907924 with nine SRA accession numbers.

## References

[1] Aatsinki A-K, Lahti L, Uusitupa H-M, Munukka E, Keskitalo A, Nolvi S, O'Mahony S, Pietilä S, Elo LL, Eerola E, Karlsson H, Karlsson L. 2019. Gut microbiota composition is associated with temperament traits in infants. Brain, Behavior, and Immunity 80:849–858. doi:10.1016/j.bbi.2019.05.035. 31132457

[2] Burleigh MC, Liddle L, Monaghan C, Muggeridge DJ, Sculthorpe N, Butcher JP, Henriquez FL, Allen JD, Easton C. 2018. Salivary nitrite production is elevated in individuals with a higher abundance of oral nitrate-reducing bacteria. Free Radical Biology and Medicine 120:80–88. doi:10.1016/j.freeradbiomed.2018.03.023. 29550328

[3] Duncan C, Dougall H, Johnston P, Green S, Brogan R, Leifert C, Smith L, Golden M, Benjamin N. 1995. Chemical generation of nitric oxide in the mouth from the enterosalivary circulation of dietary nitrate. Nat Med 1:546–551. doi:10.1038/nm0695-546. 7585121

[4] Lundberg JO, Weitzberg E, Cole JA, Benjamin N. 2004. Nitrate, bacteria and human health. Nat Rev Microbiol 2:593–602. doi:10.1038/nrmicro929. 15197394

[5] Scheiman J, Luber JM, Chavkin TA, MacDonald T, Tung A, Pham L-D, Wibowo MC, Wurth RC, Punthambaker S, Tierney BT, Yang Z, Hattab MW, Avila-Pacheco J, Clish CB, Lessard S, Church GM, Kostic AD. 2019. Meta-omics analysis of elite athletes identifies a performance-enhancing microbe that functions via lactate metabolism. Nat Med 25:1104–1109. doi:10.1038/s41591-019-0485-4. 31235964 PMC7368972

[6] Wicaksono DP, Washio J, Abiko Y, Domon H, Takahashi N. 2020. Nitrite production from nitrate and its link with lactate metabolism in oral *Veillonella* spp. Appl Environ Microbiol 86:e01255-20. doi:10.1128/AEM.01255-20. 32769185 PMC7531945

[7] Motiani KK, Collado MC, Eskelinen J-J, Virtanen KA, Löyttyniemi E, Salminen S, Nuutila P, Kalliokoski KK, Hannukainen JC. 2020. Exercise training modulates gut microbiota profile and improves endotoxemia. Med Sci Sports Exerc 52:94–104. doi:10.1249/MSS.0000000000002112. 31425383 PMC7028471

[8] Strati F, Cavalieri D, Albanese D, De Felice C, Donati C, Hayek J, Jousson O, Leoncini S, Renzi D, Calabrò A, De Filippo C. 2017. New evidences on the altered gut microbiota in autism spectrum disorders. Microbiome 5:24. doi:10.1186/s40168-017-0242-1. 28222761 PMC5320696

[9] Arrieta M-C, Stiemsma LT, Dimitriu PA, Thorson L, Russell S, Yurist-Doutsch S, Kuzeljevic B, Gold MJ, Britton HM, Lefebvre DL, Subbarao P, Mandhane P, Becker A, McNagny KM, Sears MR, Kollmann T, Mohn WW, Turvey SE, Brett Finlay B, the CHILD Study Investigators. 2015. Early infancy microbial and metabolic alterations affect risk of childhood asthma. Sci Transl Med 7:307ra152–307ra152. doi:10.1126/scitranslmed.aab2271. 26424567

[10] Wei Y, Li Y, Yan L, Sun C, Miao Q, Wang Q, Xiao X, Lian M, Li B, Chen Y, Zhang J, Li Y, Huang B, Li Y, Cao Q, Fan Z, Chen X, Fang J-Y, Gershwin ME, Tang R, Ma X. 2020. Alterations of gut microbiome in autoimmune hepatitis. Gut 69:569–577. doi:10.1136/gutjnl-2018-317836. 31201284

[11] Johns AT. 1951. The mechanism of propionic acid formation by *Veillonella gazogenes*. J General Microbiology 5:326–336. doi:10.1099/00221287-5-2-326. 14832421

[12] Distler W, Kröncke A. 1981. The lactate metabolism of the oral bacterium *Veillonella* from human saliva. Arch Oral Biol 26:657–661. and doi:10.1016/0003-9969(81)90162-X. 6947771

[13] Furusawa Y, Obata Y, Fukuda S, Endo TA, Nakato G, Takahashi D, Nakanishi Y, Uetake C, Kato K, Kato T, Takahashi M, Fukuda NN, Murakami S, Miyauchi E, Hino S, Atarashi K, Onawa S, Fujimura Y, Lockett T, Clarke JM, Topping DL, Tomita M, Hori S, Ohara O, Morita T, Koseki H, Kikuchi J, Honda K, Hase K, Ohno H. 2013. Commensal microbe-derived butyrate induces the differentiation of colonic regulatory T cells. Nature 504:446–450. doi:10.1038/nature12721. 24226770

[14] Smith PM, Howitt MR, Panikov N, Michaud M, Gallini CA, Bohlooly-Y M, Glickman JN, Garrett WS. 2013. The microbial metabolites, short-chain fatty acids, regulate colonic T_reg_ cell homeostasis. Science 341:569–573. doi:10.1126/science.1241165. 23828891 PMC3807819

[15] Chambers ES, Viardot A, Psichas A, Morrison DJ, Murphy KG, Zac-Varghese SEK, MacDougall K, Preston T, Tedford C, Finlayson GS, Blundell JE, Bell JD, Thomas EL, Mt-Isa S, Ashby D, Gibson GR, Kolida S, Dhillo WS, Bloom SR, Morley W, Clegg S, Frost G. 2015. Effects of targeted delivery of propionate to the human colon on appetite regulation, body weight maintenance and adiposity in overweight adults. Gut 64:1744–1754. doi:10.1136/gutjnl-2014-307913. 25500202 PMC4680171

[16] De Vadder F, Kovatcheva-Datchary P, Goncalves D, Vinera J, Zitoun C, Duchampt A, Bäckhed F, Mithieux G. 2014. Microbiota-generated metabolites promote metabolic benefits via gut-brain neural circuits. Cell 156:84–96. 24412651 10.1016/j.cell.2013.12.016

[17] Breton J, Tennoune N, Lucas N, Francois M, Legrand R, Jacquemot J, Goichon A, Guérin C, Peltier J, Pestel-Caron M, Chan P, Vaudry D, do Rego J-C, Liénard F, Pénicaud L, Fioramonti X, Ebenezer IS, Hökfelt T, Déchelotte P, Fetissov SO. 2016. Gut commensal *E. coli* proteins activate host satiety pathways following nutrient-induced bacterial growth. Cell Metabolism 23:324–334. doi:10.1016/j.cmet.2015.10.017. 26621107

[18] Li G, Xie C, Lu S, Nichols RG, Tian Y, Li L, Patel D, Ma Y, Brocker CN, Yan T, Krausz KW, Xiang R, Gavrilova O, Patterson AD, Gonzalez FJ. 2017. Intermittent fasting promotes white adipose browning and decreases obesity by shaping the gut microbiota. Cell Metabolism 26:672–685.e4. doi:10.1016/j.cmet.2017.08.019. 28918936 PMC5668683

[19] Shi H, Zhang B, Abo-Hamzy T, Nelson JW, Ambati CSR, Petrosino JF, Bryan RM, Durgan DJ. 2021. Restructuring the gut microbiota by intermittent fasting lowers blood pressure. Circ Res 128:1240–1254. doi:10.1161/CIRCRESAHA.120.318155. 33596669 PMC8085162

[20] Guo Y, Luo S, Ye Y, Yin S, Fan J, Xia M. 2021. Intermittent fasting improves cardiometabolic risk factors and alters gut microbiota in metabolic syndrome patients. J Clin Endocrinol Metab 106:64–79. doi:10.1210/clinem/dgaa644. 33017844

[21] Liu Z, Dai X, Zhang H, Shi R, Hui Y, Jin X, Zhang W, Wang L, Wang Q, Wang D, Wang J, Tan X, Ren B, Liu X, Zhao T, Wang J, Pan J, Yuan T, Chu C, Lan L, Yin F, Cadenas E, Shi L, Zhao S, Liu X. 2020. Gut microbiota mediates intermittent-fasting alleviation of diabetes-induced cognitive impairment. Nat Commun 11:855. doi:10.1038/s41467-020-14676-4. 32071312 PMC7029019

[22] Nyström T. 2004. Stationary-phase physiology. Annu Rev Microbiol 58:161–181. doi:10.1146/annurev.micro.58.030603.123818. 15487934

[23] Navarro Llorens JM, Tormo A, Martínez-García E. 2010. Stationary phase in Gram-negative bacteria. FEMS Microbiol Rev 34:476–495. and doi:10.1111/j.1574-6976.2010.00213.x. 20236330

[24] Zimmer DP, Soupene E, Lee HL, Wendisch VF, Khodursky AB, Peter BJ, Bender RA, Kustu S. 2000. Nitrogen regulatory protein C-controlled genes of *Escherichia coli*: scavenging as a defense against nitrogen limitation. Proc Natl Acad Sci USA 97:14674–14679. doi:10.1073/pnas.97.26.14674. 11121068 PMC18977

[25] Glasser NR, Kern SE, Newman DK. 2014. Phenazine redox cycling enhances anaerobic survival in *Pseudomonas aeruginosa* by facilitating generation of ATP and a proton-motive force. Mol Microbiol 92:399–412. doi:10.1111/mmi.12566. 24612454 PMC4046897

[26] Eschbach M, Schreiber K, Trunk K, Buer J, Jahn D, Schobert M. 2004. Long-term anaerobic survival of the opportunistic pathogen *Pseudomonas aeruginosa* via pyruvate fermentation. J Bacteriol 186:4596–4604. doi:10.1128/JB.186.14.4596-4604.2004. 15231792 PMC438635

[27] Sato N, Kakuta M, Hasegawa T, Yamaguchi R, Uchino E, Kobayashi W, Sawada K, Tamura Y, Tokuda I, Murashita K, Nakaji S, Imoto S, Yanagita M, Okuno Y. 2020. Metagenomic analysis of bacterial species in tongue microbiome of current and never smokers. NPJ Biofilms Microbiomes 6:11. doi:10.1038/s41522-020-0121-6. 32170059 PMC7069950

[28] Wang L, Yu X, Xu X, Ming J, Wang Z, Gao B, Xing Y, Zhou J, Fu J, Liu T, Liu X, Garstka MA, Wang X, Ji Q. 2021. The fecal microbiota is already altered in normoglycemic individuals who go on to have type 2 diabetes. Front Cell Infect Microbiol 11:598672. doi:10.3389/fcimb.2021.598672. 33680988 PMC7930378

[29] Caspi R, Altman T, Billington R, Dreher K, Foerster H, Fulcher CA, Holland TA, Keseler IM, Kothari A, Kubo A, Krummenacker M, Latendresse M, Mueller LA, Ong Q, Paley S, Subhraveti P, Weaver DS, Weerasinghe D, Zhang P, Karp PD. 2014. The MetaCyc database of metabolic pathways and enzymes and the BioCyc collection of pathway/genome databases. Nucleic Acids Res 42:D459–D471. doi:10.1093/nar/gkt1103. 24225315 PMC3964957

[30] Seeliger S, Janssen PH, Schink B. 2002. Energetics and kinetics of lactate fermentation to acetate and propionate via methylmalonyl-CoA or acrylyl-CoA. FEMS Microbiol Lett 211:65–70. doi:10.1111/j.1574-6968.2002.tb11204.x. 12052552

[31] Michaud R, Delwiche E. 1970. Multiple impairment of glycolysis in Veillonella *alcalescens*. J Bacteriol 101:138–140. doi:10.1128/jb.101.1.138-140.1970. 5460841 PMC250461

[32] Ng SK, Hamilton IR. 1974. Gluconeogenesis by *Veillonella parvula* M4: evidence for the indirect conversion of pyruvate to P-enolpyruvate. Can J Microbiol 20:19–28. doi:10.1139/m74-004. 4822778

[33] Louis P, Flint HJ. 2017. Formation of propionate and butyrate by the human colonic microbiota. Environ Microbiol 19:29–41. doi:10.1111/1462-2920.13589. 27928878

[34] Hilpert W, Dimroth P. 1983. Purification and characterization of a new sodium-transport decarboxylase: methytlaomyl-CoA decarboxylase from *Veillonella alcalescens*. Eur J Biochem 132:579–587. doi:10.1111/j.1432-1033.1983.tb07403.x. 6852015

[35] Reeve C, Amy P, Matin A. 1984. Role of protein synthesis in the survival of carbon-starved *Escherichia coli* K-12. J Bacteriol 160:1041–1046. doi:10.1128/jb.160.3.1041-1046.1984. 6389505 PMC215816

[36] Thaiss CA, Zeevi D, Levy M, Zilberman-Schapira G, Suez J, Tengeler AC, Abramson L, Katz MN, Korem T, Zmora N, Kuperman Y, Biton I, Gilad S, Harmelin A, Shapiro H, Halpern Z, Segal E, Elinav E. 2014. Transkingdom control of microbiota diurnal oscillations promotes metabolic homeostasis. Cell 159:514–529. 25417104 10.1016/j.cell.2014.09.048

[37] Nencioni A, Caffa I, Cortellino S, Longo VD. 2018. Fasting and cancer: molecular mechanisms and clinical application. Nat Rev Cancer 18:707–719. doi:10.1038/s41568-018-0061-0. 30327499 PMC6938162

[38] Mattson MP, Longo VD, Harvie M. 2017. Impact of intermittent fasting on health and disease processes. Ageing Res Rev 39:46–58. doi:10.1016/j.arr.2016.10.005. 27810402 PMC5411330

[39] Johnson JB, Summer W, Cutler RG, Martin B, Hyun D-H, Dixit VD, Pearson M, Nassar M, Telljohann R, Maudsley S, Carlson O, John S, Laub DR, Mattson MP. 2007. Alternate day calorie restriction improves clinical findings and reduces markers of oxidative stress and inflammation in overweight adults with moderate asthma. Free Radic Biol Med 42:665–674. doi:10.1016/j.freeradbiomed.2006.12.005. 17291990 PMC1859864

[40] Morita C, Tsuji H, Hata T, Gondo M, Takakura S, Kawai K, Yoshihara K, Ogata K, Nomoto K, Miyazaki K, Sudo N. 2015. Gut dysbiosis in patients with anorexia nervosa. PLoS One 10:e0145274. doi:10.1371/journal.pone.0145274. 26682545 PMC4687631

[41] Li L, Su Y, Li F, Wang Y, Ma Z, Li Z, Su J. 2020. The effects of daily fasting hours on shaping gut microbiota in mice. BMC Microbiol 20:65. doi:10.1186/s12866-020-01754-2. 32209070 PMC7092480

[42] Mays TD, Holdeman LV, Moore WEC, Rogosa M, Johnson JL. 1982. Taxonomy of the genus *Veillonella* Prévot. Int J Systematic Bacteriology 32:28–36. doi:10.1099/00207713-32-1-28.

[43] De Baere S, Eeckhaut V, Steppe M, De Maesschalck C, De Backer P, Van Immerseel F, Croubels S. 2013. Development of a HPLC–UV method for the quantitative determination of four short-chain fatty acids and lactic acid produced by intestinal bacteria during in vitro fermentation. J Pharm Biomed Anal 80:107–115. doi:10.1016/j.jpba.2013.02.032. 23542733

[44] Faijes M, Mars AE, Smid EJ. 2007. Comparison of quenching and extraction methodologies for metabolome analysis of *Lactobacillus plantarum*. Microb Cell Fact 6:27. doi:10.1186/1475-2859-6-27. 17708760 PMC2031893

[45] Ge SX, Son EW, Yao R. 2018. iDEP: an integrated web application for differential expression and pathway analysis of RNA-Seq data. BMC Bioinformatics 19:534. doi:10.1186/s12859-018-2486-6. 30567491 PMC6299935

[46] Xie C, Mao X, Huang J, Ding Y, Wu J, Dong S, Kong L, Gao G, Li C-Y, Wei L. 2011. KOBAS 2.0: a web server for annotation and identification of enriched pathways and diseases. Nucleic Acids Res 39:W316–W322. doi:10.1093/nar/gkr483. 21715386 PMC3125809

