# Supplementary material for: The Commensal Anaerobe Veillonella dispar Reprograms Its Lactate Metabolism and Short-Chain Fatty Acid Production during the Stationary Phase
Source: Microbiol Spectr. 2023 Mar 28;11(2):e03558-22. doi: 10.1128/spectrum.03558-22 (PMC10100942; doi:10.1128/spectrum.03558-22)
Supplement: Supplemental file 1 — Supplemental material. Download spectrum.03558-22-s0001.pdf, PDF file, 0.7 MB [file spectrum.03558-22-s0001.pdf]

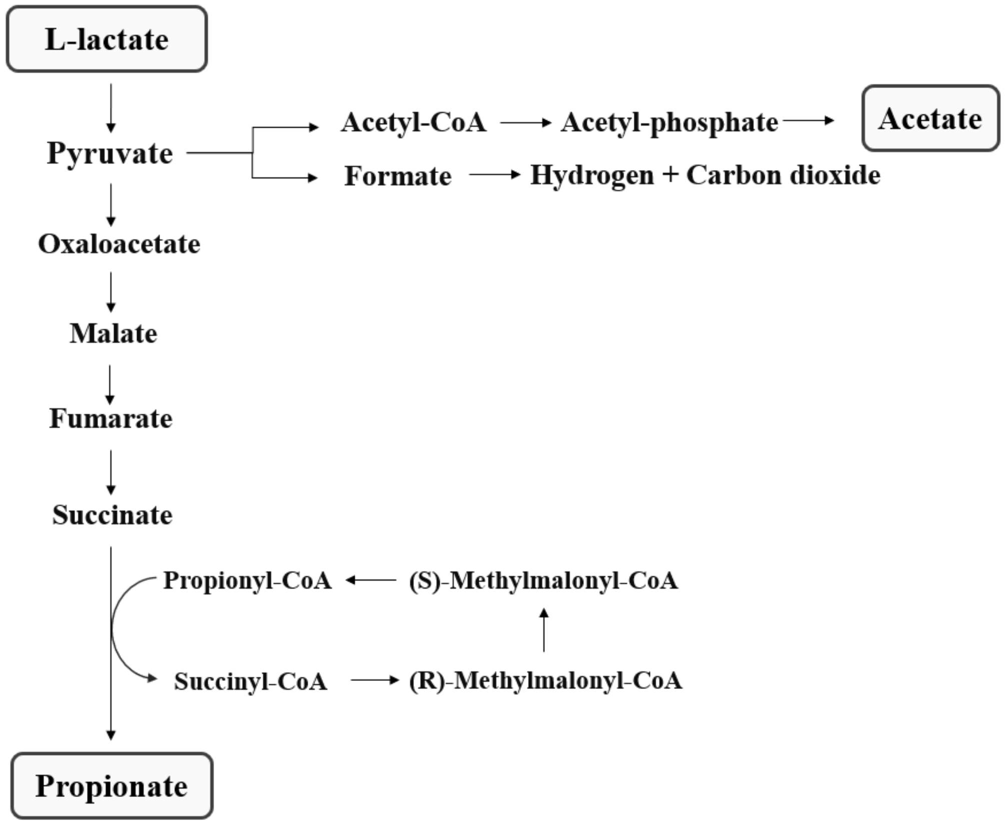

Figure S1. Proposed pathway for L-lactate metabolism by *V. dispar*

The proposed lactate metabolic pathway was constructed based on information available from the KEGG pathway database, the MetaCyc database, and the reference genome of *V. dispar* ATCC 17748. Lactate is oxidized to pyruvate with being NADH produced. The produced pyruvate is further metabolized with two main metabolic fluxes. In the flux to acetate production, pyruvate is catabolized to formate and acetyl-coA by pyruvate-formate lyase. The acetyl-coA is further catalyzed to acetyl-phosphate and finally to acetate. The formate is metabolized to hydrogen. In the flux to propionate production, the pyruvate is converted to oxaloacetate by pyruvate carboxylase, followed by serial reactions of the reversed TCA cycle pathway. Oxaloacetate is catalyzed to malate, fumarate and succinate sequentially. Succinate and propionyl-CoA are then converted to propionate and succinate-CoA by propionyl-CoA: succinate CoA transferase. Succinyl-CoA is recycled to propionyl-CoA by methylmalonyl-CoA mutase, methylmalonyl-CoA epimerase, and methylmalonyl-CoA decarboxylase.

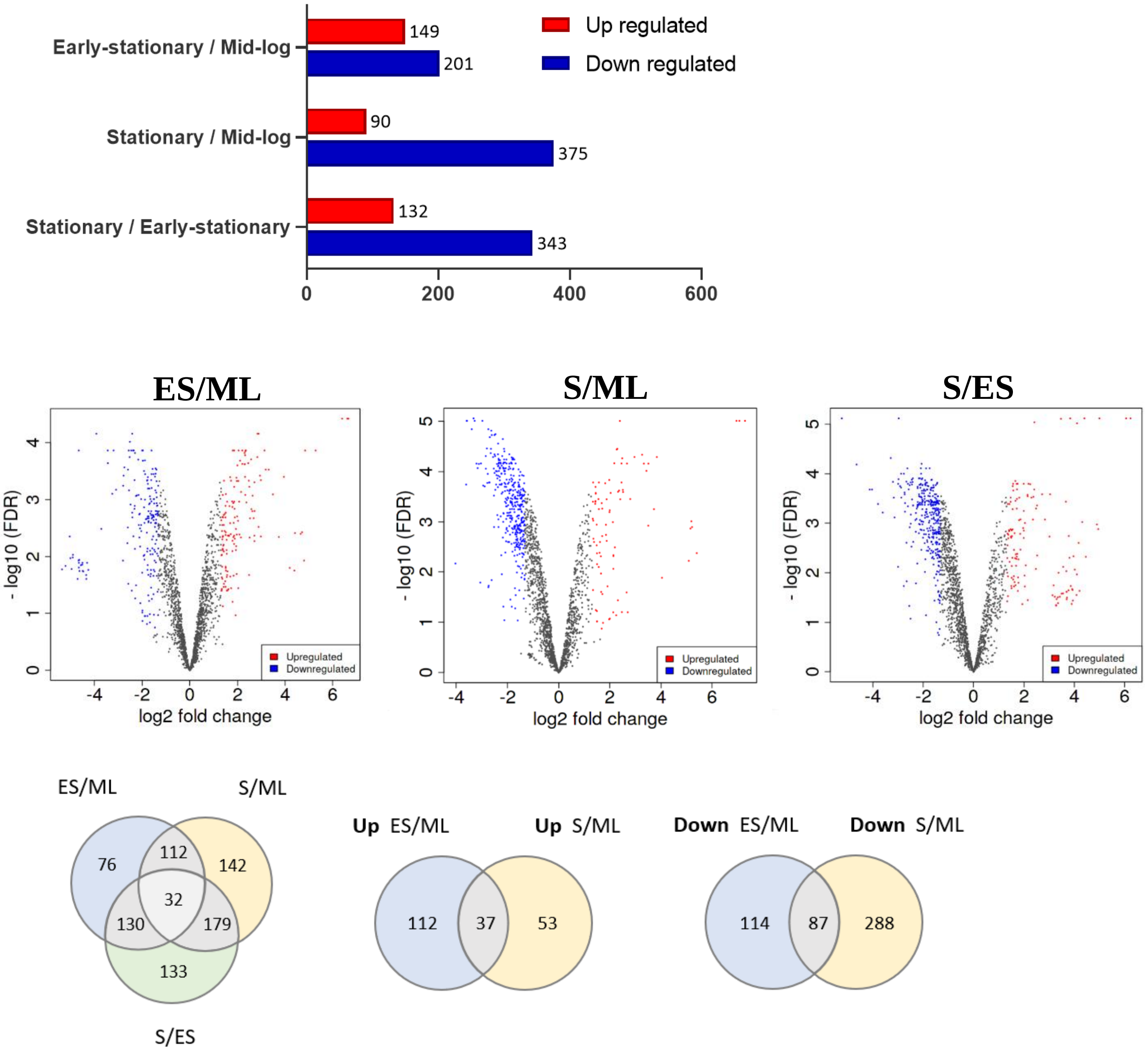

Figure S2. Differentially expressed gene (DEG) analysis

(A) Bar graph of DEGs of the three comparison groups. **(B)** Volcano plots show the different gene expression patterns between the two selected growth phases. The red dots represent significantly up regulated genes and blue dots the significantly down regulated gene (absolute log2 fold change >1 and adjusted $p < 0.01$); black dots represent the gene not significantly regulated. **(C)** Venn diagram of DEGs under different comparison conditions. ML, Mid-log; ES, Early-stationary; S, Stationary; ES/ML means ES versus ML.

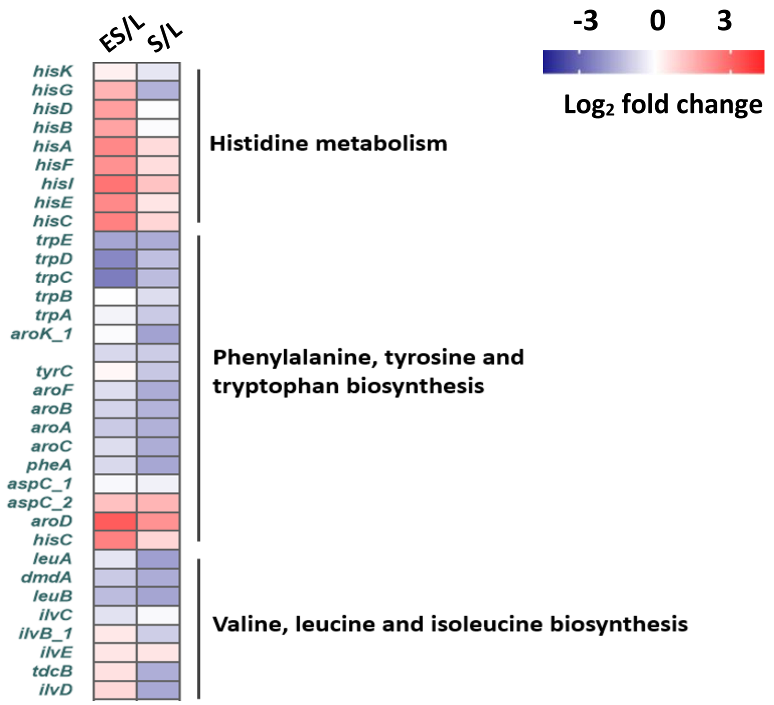

Figure S3. Differentially expressed amino acid metabolism/biosynthesis pathway

Heatmap of genes in differentially expressed amino acid metabolism/biosynthesis pathways during the growth are shown. The color key indicates transcript enrichment (red) and depletion (blue). Values represent the log_2 fold change of transcript level between compared conditions. ES/L, Early stationary phase versus log phase; S/L, stationary phase versus log phase.

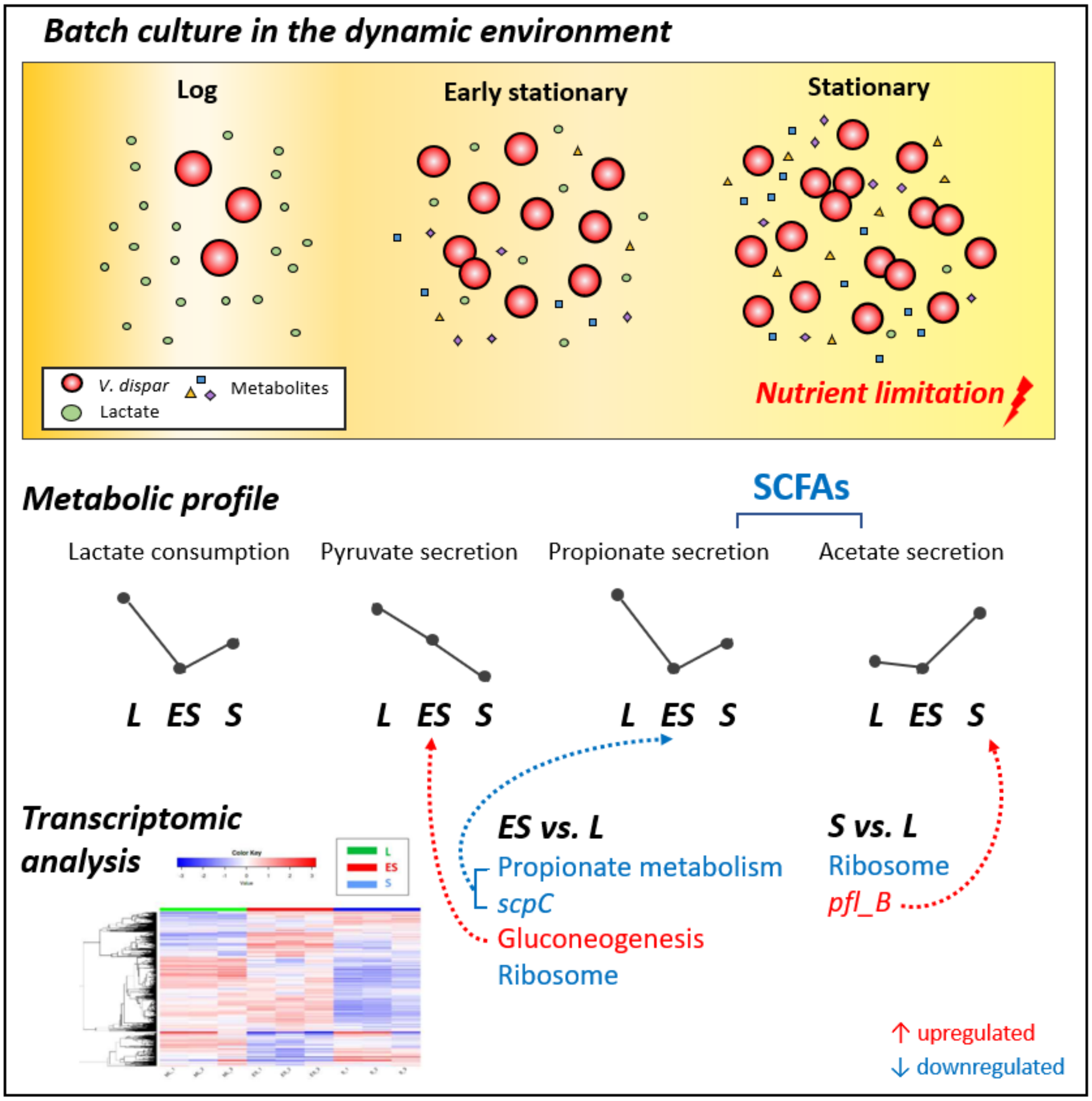

Figure S4 | Alterations of metabolic profile and gene expression of V. dispar during the anaerobic growth

V. dispar was found to have an altered lactate metabolic profile and changes in gene expression in various growth phases: acetate production was increased, and lactate consumption and propionate production were both decreased during early stationary phase, and these were partially restored during stationary phase. *V. dispar* had distinct transcriptomes across the three growth phases. The gene *pfl_B* was upregulated during stationary phase, which could then direct the flow of carbon metabolism into acetate production and this may explain the increased acetate production and

increased intracellular acetate level during stationary phase. Propionate metabolism was downregulated during early stationary phase, which seems to correlate with the decreased propionate production during early stationary phase. Gluconeogenesis was upregulated during early stationary phase, and this may provide an alternative metabolic route for pyruvate utilization during early stationary phase and stationary phase.

Table S1. Transcript level of seleceted genes

ID (KEGG)	Gene	Log$_2$ fold change		Product
		ES /ML	***S/ML***[a]	
Propionate metabolism				
NCTC11831_01812	*yqhD*	-1.44*[b]	-0.02	NADP-dependent alcohol dehydrogenase
NCTC11831_01756	-	-4.63*	-0.92	Diol dehydratase subunit alpha
NCTC11831_01759	*pduC*	-4.34*	-0.64	Propanediol dehydratase large subunit
NCTC11831_01758	*pduD*	-4.96*	-0.75	Propanediol dehydratase medium subunit
NCTC11831_01757	*pduE*	-4.65*	-0.72	Propanediol dehydratase small subunit
NCTC11831_01740	*sucD*	-4.50*	-1.16	Propionaldehyde dehydrogenase
NCTC11831_00834	*ppsA*	1.64*	-1.09	Phosphoenolpyruvate synthase
Acetate metabolism				
NCTC11831_00242	pflB_2	0.60	3.32*	Formate acetyltransferase
NCTC11831_01878	eutD	-0.21	-0.15	Phosphate acetyltransferase
NCTC11831_00645	ackA	-0.42	-1.01	Acetate kinase
Gluconeogenesis				
NCTC11831_00834	*ppsA*	1.64*	-1.09*	Phosphoenolpyruvate synthase
NCTC11831_00807	*eno*	1.86*	0.493	Enolase
NCTC11831_00806	*gpmI*	1.77*	0.626	2,3-bisphosphoglycerate-independent phosphoglycerate mutase
NCTC11831_00804	*pgk*	1.50*	0.774*	Phosphoglycerate kinase
NCTC11831_00803	*gap*	1.32*	0.923*	Glyceraldehyde 3-phosphate dehydrogenase
NCTC11831_00805	*tpiA*	1.66*	0.676*	Triosephosphate isomerase
Biotin metabolism				
NCTC11831_00200	*bioB*	4.19*	4.03*	Biotin synthase
NCTC11831_00221	*bioD*	4.39*	5.08*	Dethiobiotin synthetase
NCTC11831_00222	*bioK*	4.79*	5.39*	Adenosylmethionine---8-amino-7-oxononanoate aminotransferase
NCTC11831_00892	*bioF*	2.98*	3.50*	8-amino-7-oxononanoate synthase
NCTC11831_00891	*bioW*	1.56*	0.06	6-carboxyhexanoate--coa ligase
NCTC11831_00257	*bioY*	1.29*	1.50*	Histidinol-phosphatase
Two-component system				
NCTC11831_01511	*citD*	1.40*	0.56	Citrate lyase subunit gamma
NCTC11831_01510	*citE*	1.59*	0.60	Citrate lyase subunit beta
NCTC11831_01509	*citF*	1.72*	0.39	Citrate lyase subunit alpha
Nitrogen metabolism				
NCTC11831_01630	*norB*	2.47*	2.47*	Nitric oxide reductase subunit B
NCTC11831_01056	*glnA*	1.56*	0.06	Glutamine synthetase
NCTC11831_01804	*gltB*	4.84*	0.80	Ferredoxin-dependent glutamate synthase 1
NCTC11831_00207	*narK*	1.46*	0.37	Nitrite facilitator 1

Histidine metabolism

NCTC11831_00121	*hisG*	1.46*	-1.51*	ATP phosphoribosyltransferase
NCTC11831_00122	*hisD*	1.87*	-0.05	Histidinol dehydrogenase
NCTC11831_00123	*hisB*	1.78*	-0.08	Imidazoleglycerol-phosphate dehydratase
NCTC11831_00124	*hisA*	2.31*	0.69	Phosphoribosylformimino-5-aminoimidazole carboxamide ribotide isomerase
NCTC11831_00125	*hisF*	2.15*	0.66	Imidazole glycerol-phosphate synthase subunit hisf
NCTC11831_00126	-	2.71*	1.15*	Phosphoribosyl-AMP cyclohydrolase
NCTC11831_00127	*hisE*	2.30*	0.50	Phosphoribosyl-ATP pyrophosphohydrolase
NCTC11831_00128	*hisC*	2.45*	0.79	Histidinol-phosphate aminotransferase
NCTC11831_00129	*hisH*	1.69*	1.01*	Imidazole glycerol-phosphate synthase subunit hish
NCTC11831_00130	*hisF*	1.43*	0.91	Imidazole glycerol-phosphate synthase subunit hisf
NCTC11831_00119	*hisK*	0.28	-0.50	Histidinol-phosphata

Phenylalanine, tyrosine and tryptophan biosynthesis

NCTC11831_01779	*aroA*	-1.04	-1.53*	3-phosphoshikimate 1-carboxyvinyltransferase
NCTC11831_01778	*aroB*	-0.86	-1.47*	3-dehydroquinate synthase
NCTC11831_01780	*aroC*	-0.68	-1.61*	Chorismate synthase
NCTC11831_01784	*aroD*	3.18*	2.11*	3-dehydroquinate dehydratase
NCTC11831_01782	*aroE*	-1.18*	-1.52*	Shikimate dehydrogenase
NCTC11831_01777	*aroF*	-0.64	-1.63*	3-deoxy-7-phosphoheptulonate synthase
NCTC11831_00820	*aroK*	-0.09	-1.82*	Shikimate kinase
NCTC11831_00788	*aspC*	1.20*	1.43*	Aromatic-amino-acid transaminase
NCTC11831_00056	*aspC*	-0.15	-0.29	Aspartate aminotransferase
NCTC11831_00128	*hisC*	2.45*	0.79*	Histidinol-phosphate aminotransferase
NCTC11831_01781	*pheA*	-0.75	-1.74*	Chorismate mutase / prephenate dehydratase
NCTC11831_00164	*trpA*	-0.24	-1.04*	Tryptophan synthase alpha chain
NCTC11831_00163	*trpB*	-0.02	-0.67*	Tryptophan synthase beta chain
NCTC11831_00162	*trpC*	-2.56*	-1.32*	Indole-3-glycerol phosphate synthase
NCTC11831_00161	*trpD*	-2.35*	-1.25*	Anthranilate phosphoribosyltransferase
NCTC11831_00159	*trpE*	-1.75*	-1.64*	Anthranilate synthase component I
NCTC11831_01574	*tyrC*	0.14	-1.10*	Prephenate dehydrogenase
NCTC11831_01048	-	-0.78	-1.00*	Chorismate mutase

Threonine, valine, leucine and isoleucine biosynthesis

NCTC11831_00084	*leuA*	-0.51	-1.88*	2-isopropylmalate synthase
NCTC11831_00085	*dmdA*	-1.05*	-1.63*	(R)-2-methylmalate dehydratase large subunit
NCTC11831_00086	*DmdB*	-1.07*	-1.72*	(R)-2-methylmalate dehydratase small subunit
NCTC11831_00087	*leuB*	-1.33*	-1.78*	3-isopropylmalate dehydrogenase
NCTC11831_00508	*ilvB*	0.45	-0.96*	Acetolactate synthase I/II/III large subunit
NCTC11831_00509	*ilvH*	0.13	-1.08*	Acetolactate synthase I/III small subunit
NCTC11831_01203	*ilvE*	0.49	0.49	Branched-chain amino acid aminotransferase
NCTC11831_01543	*ilvC*	-0.57*	-0.13	Ketol-acid reductoisomerase
NCTC11831_01545	*ilvH*	0.24	-2.15*	Acetolactate synthase I/III small subunit
NCTC11831_01546	*ilvB*	0.35	-1.87*	Acetolactate synthase I/II/III large subunit
NCTC11831_01547	*tdcB*	0.57	-1.59*	Threonine dehydratase
NCTC11831_01548	*ilvD*	0.74*	-1.70*	Dihydroxy-acid dehydratase
NCTC11831_00765	*lysC*	0.53	-1.08*	Aspartate kinase

NCTC11831_01016	*asd*	0.14	-0.56*	Aspartate-semialdehyde dehydrogenase
NCTC11831_01047	*hom*	-0.95*	-1.27*	Homoserine dehydrogenase
NCTC11831_01046	*thrB*	-1.08*	-1.41*	Homoserine kinase
NCTC11831_00594	*thrC*	0.78	-0.19	Threonine synthase
Ribosome				
NCTC11831_00659	*rplU*	-0.76	-2.34*	large subunit ribosomal protein L21
NCTC11831_00661	*rpmA*	-1.27*	-2.18*	large subunit ribosomal protein L27
NCTC11831_00838	*rpmB*	-1.97*	-2.63*	large subunit ribosomal protein L28
NCTC11831_00971	*rplS*	-1.21*	-2.37*	large subunit ribosomal protein L19
NCTC11831_01089	*rpsB*	-0.88	-2.97*	small subunit ribosomal protein S2
NCTC11831_01180	*rpsO*	-1.19*	-2.61*	small subunit ribosomal protein S15
NCTC11831_01556	*rpsI*	-1.43*	-3.32*	small subunit ribosomal protein S9
NCTC11831_01557	*rplM*	-1.15*	-3.28*	large subunit ribosomal protein L13
NCTC11831_01586	*rpsK*	-1.05*	-2.27*	small subunit ribosomal protein S11
NCTC11831_01587	*rpsM*	-1.08*	-2.16*	small subunit ribosomal protein S13
NCTC11831_01588	*rpmJ*	-1.73*	-3.11*	large subunit ribosomal protein L36
NCTC11831_01593	*rplO*	-1.34*	-2.11*	large subunit ribosomal protein L15
NCTC11831_01594	*rpmD*	-1.79*	-2.22*	large subunit ribosomal protein L30
NCTC11831_01595	*rpsE*	-1.27*	-2.29*	small subunit ribosomal protein S5
NCTC11831_01596	*rplR*	-1.33*	-2.34*	large subunit ribosomal protein L18
NCTC11831_01597	*rplF*	-1.37*	-2.33*	large subunit ribosomal protein L6
NCTC11831_01598	*rpsH*	-1.37*	-2.34*	small subunit ribosomal protein S8
NCTC11831_01600	*rplE*	-1.43*	-2.36*	large subunit ribosomal protein L5
NCTC11831_01601	*rplX*	-1.41*	-2.33*	large subunit ribosomal protein L24
NCTC11831_01602	*rplN*	-1.60*	-2.40*	large subunit ribosomal protein L14
NCTC11831_01603	*rpsQ*	-1.50*	-2.41*	small subunit ribosomal protein S17
NCTC11831_01604	*rpmC*	-2.25*	-2.00*	large subunit ribosomal protein L29
NCTC11831_01605	*rplP*	-1.61*	-2.45*	large subunit ribosomal protein L16
NCTC11831_01606	*rpsC*	-1.45*	-2.36*	small subunit ribosomal protein S3
NCTC11831_01607	*rplV*	-1.53*	-2.30*	large subunit ribosomal protein L22
NCTC11831_01608	*rpsS*	-1.65*	-2.39*	small subunit ribosomal protein S19
NCTC11831_01609	*rplB*	-1.57*	-2.24*	large subunit ribosomal protein L2
NCTC11831_01610	*rplW*	-1.72*	-2.44*	large subunit ribosomal protein L23
NCTC11831_01611	*rplD*	-1.59*	-2.30*	large subunit ribosomal protein L4
NCTC11831_01612	*rplC*	-1.58*	-2.29*	large subunit ribosomal protein L3
NCTC11831_01613	*rpsJ*	-1.63*	-2.37*	small subunit ribosomal protein S10
NCTC11831_01623	*rplL*	-1.87*	-3.58*	large subunit ribosomal protein L7/L12
NCTC11831_01624	*rplJ*	-1.49*	-3.59*	large subunit ribosomal protein L10
NCTC11831_01625	*rplA*	-1.51*	-2.65*	large subunit ribosomal protein L1
NCTC11831_01626	*rplK*	-1.49*	-2.56*	large subunit ribosomal protein L11

- [a] The log_2 fold change of transcript level of genes in early-stationary (*ES*) phase and stationary (*S*)
- phase groups relative to mid-log (*ML*) phase. "*S/ML*" means *S* vs. *ML*. *, p < 0.01.
